# Spontaneous Coronary Artery Dissection and a Family History of Aortic Dissection: A Genetic Association Study

**DOI:** 10.1161/JAHA.124.037921

**Published:** 2025-04-07

**Authors:** Lucy McGrath‐Cadell, Stephanie Hesselson, Ingrid Tarr, Emma M. Rath, Michael Troup, Yunkai Gao, Keerat Junday, Monique Bax, Siiri E. Iismaa, Nicholas Collins, David W. M. Muller, Jason C. Kovacic, Eleni Giannoulatou, Robert M. Graham

**Affiliations:** ^1^ Victor Chang Cardiac Research Institute, Darlinghurst Sydney Australia; ^2^ School of Clinical Medicine, St Vincent’s Healthcare Clinical Campus, Faculty of Medicine and Health UNSW Sydney Australia; ^3^ UNSW Sydney Kensington Australia; ^4^ Cardiology Department John Hunter Hospital New Lambton Heights Australia; ^5^ Cardiology Department St Vincent’s Hospital Darlinghurst Australia; ^6^ Cardiovascular Research Institute Icahn School of Medicine at Mount Sinai New York NY USA

**Keywords:** spontaneous coronary artery dissection, aortic dissection, whole genome sequencing, pathogenic variants, polygenic risk score, Genetics

## Abstract

**Background:**

Spontaneous coronary artery dissection (SCAD) is an increasingly recognized cause of acute coronary syndrome or sudden cardiac death, primarily affecting relatively young women (median age, 51 years) without typical cardiovascular risk factors. SCAD has a genetic component, with genome‐wide association studies identifying multiple risk loci. Thoracic aortic dissection (type A) shares some genetic overlap with SCAD, suggesting potential common predispositions.

**Methods:**

We performed genetic screening or whole‐genome sequencing of 17 patients with SCAD (94% women) with a first‐ or second‐degree relative (89% men) affected by aortic dissection (AD). We assessed rare variants in candidate genes and genome‐wide using the American College of Medical Genetics and Genomics criteria. Polygenic risk scores were calculated to assess genetic risk for SCAD, fibromuscular dysplasia, AD, and abdominal aortic aneurysm in patients with SCAD, relatives with AD, and controls.

**Results:**

Whole‐genome sequencing identified pathogenic or likely pathogenic variants in *SMAD3*, *CBS*, and *COL3A1* in 3 SCAD cases. Additionally, 4 variants of uncertain significance were found in candidate genes. Polygenic risk scores for SCAD were significantly associated with increased odds of SCAD in probands versus controls (odds ratio, 1.79 [95% CI, 1.08–2.99]; *P*=0.024).

**Conclusions:**

Our study supports a complex genetic landscape underlying SCAD, implicating rare monogenic pathogenic variants and polygenic risk. We identified pathogenic variants in patients with SCAD with a family history of AD, highlighting potential genetic links between these vascular disorders. The findings underscore the importance of genetic screening in patients with SCAD with a history of AD to identify individuals at risk and guide preventive strategies.

**Nonstandard Abbreviations and Acronyms**
AD: aortic dissection
CTD: connective tissue disorder
FMD: fibromuscular dysplasia
PRS: polygenic risk score
SCAD: spontaneous coronary artery dissection
VUS: variant of uncertain significance
WGS: whole‐genome sequencing

**Clinical Perspective**

**What Is New?**

- In an angiographically confirmed spontaneous coronary artery dissection cohort of 435 participants, 5% reported a family history of aortic dissection.
- Of the 17 participants with spontaneous coronary artery dissection with a family history of aortic dissection who underwent genetic screening, 35% carried at least 1 predicted damaging variant, including 3 pathogenic/likely pathogenic variants and 4 variants of uncertain significance.

**What Are the Clinical Implications?**

- A family history of aortic dissections in patients with spontaneous coronary artery dissection should prompt consideration for clinical genetic testing referrals.

Spontaneous coronary artery dissection (SCAD) is a nonatheromatous and noninflammatory disorder that manifests clinically as an acute coronary syndrome or sudden cardiac death. It most often affects relatively young women (average age, 45–52 years),^1^ who lack traditional cardiovascular risk factors. SCAD is generally thought to be caused by an intramural bleed in the medial layer of an epicardial coronary artery wall,^2^ leading to a hematoma, that is potentially due to vasa vasorum compromise,^3^ and occurs with or without an associated tear in the endothelium. This manifests as either complete or partial occlusion of the coronary artery resulting in myocardial ischemia, infarction, or death.

SCAD is associated clinically with vascular and connective tissue disorders (CTDs), most commonly fibromuscular dysplasia (FMD) in 45% to 86% of cases, with considerable variability in diagnostic rate likely due to differences in the completeness of screening undertaken.^1^ An interaction between background genetic risk in an individual and environmental factors (e.g., physical, emotional, or hormonal stressors) is hypothesized to lead to SCAD. However, rare monogenic pathogenic variants are believed to underlie only a small proportion of SCAD cases (<4%–13%).^4^, ^5^, ^6^ Consequently, polygenic risk profiling is increasingly recognized as a valuable means of assessing genetic risk in these patients.

The first common genetic association shown in a large population of patients with SCAD was a single‐nucleotide variant (SNV) in the phosphatase and actin regular gene 1 (*PHACTR1*).^7^ The rs9349379 variant in *PHACTR1* has opposing risk associations depending on which purine (A or G) is present.^7^ The G allele associates with coronary artery disease and calcification and the A allele associates with SCAD, FMD, migraine, cervical artery dissection, and hypertension.^7^

A recent genome‐wide association study (GWAS) meta‐analysis of SCAD identified 16 common risk loci for SCAD.^8^ In general, these loci were closest upstream or downstream to genes important in extracellular matrix biology, vascular tone, and blood coagulation.^8^ Polygenic risk scores (PRSs) have been developed and validated in SCAD cohorts using common variants identified in GWASs.^9^ We recently showed, for example, that a high PRS, determined using a score computed using 7 GWAS‐determined SNVs, was associated with an increased risk of SCAD in familial as well as sporadic SCAD cases compared with controls.^10^

Thoracic aortic dissection (type A) is caused by an endothelial tear that allows blood to track between the intima and media creating a “false lumen.” The genetics of aortic dissection (AD), specifically thoracic AD in the context of ascending aortic dilatation or aneurysm, are better studied and understood than those for SCAD.^11^, ^12^, ^13^ The condition is divided into syndromic cases, with other features of CTDs, and nonsyndromic cases who lack such connective tissue features. A positive family history (familial, nonsyndromic) is found in around 20% of nonsyndromic cases of thoracic aortic aneurysm and dissection.^14^

A recent literature review identified 15 genes associated with both SCAD and thoracic AD, some of which may underlie monogenic forms of either disease and others that were implicated in a GWAS.^15^ The monogenic conditions are autosomal dominant CTDs, such as Marfan syndrome (*FBN1* mutations encoding fibrillin 1), vascular Ehlers–Danlos syndrome (*COL3A1* mutations encoding type 3 collagen) or Loeys–Dietz syndrome (*TGFBR‐1* and ‐*2* mutations encoding transforming growth factor β receptors 1 and 2, and *SMAD3* mutations).^15^

Overlap between SCAD and aortic dissection (AD) in families and individuals has been reported, albeit not commonly. For example, in a cohort of >7000 individuals with thoracic aortic disease, 0.15% were reported to have had a SCAD.^16^ Isolated case reports also document the association of SCAD and thoracic AD; for example: a 48‐year‐old woman who suffered a SCAD of the left anterior descending coronary artery had a family history significant for a mother dying suddenly from an ascending AD in her 50s^17^; a postpartum 27‐year‐old woman who presented with a left anterior descending artery SCAD and a type B AD,^18^ and a 73‐year‐old woman with a type A AD who was subsequently diagnosed with multivessel SCADs after being unable to be weaned off bypass following emergency surgery.^19^ Here, we report on familial clustering of SCAD and AD in our cohort of 435 patients with SCAD, with angiographic confirmation, and identify potential genetic associations.

## Objective

The aim of this study was to assess genetic data from patients with SCAD and relatives with AD to determine the contribution of rare and common genetic variants to the development of these diseases.

## Methods

### Data Access and Responsibility

The data that support the findings of this study are not publicly available due to patient privacy and consent requirements but are available from the corresponding author upon reasonable request and subject to the necessary legal arrangements.

### Study Cohort and Genetic Analysis

The SCAD cohort used for this genetic association study was part of the VASC (VCCRI Arteriopathies and SCAD Cohort), recruited between 2017 and 2023 via social media and specialist referral. Written, informed consent was obtained from all participants. The study has human ethics approval through the St Vincent's Hospital Human Research Ethics Committee (2019/ETH03171). Confirmation of SCAD diagnosis was made for all cases through verification of the original invasive angiogram by an expert interventional cardiologist (D.W.M.M.). Clinical data were collected for all cases through in‐depth phone interview, specialist letters, and hospital records and stored in a secure and searchable RedCap database. At the time of the phone interview or of clinical evaluation of SCAD cases seen at St Vincent's Hospital (D.W.M.M., J.C.K., R.M.G.) or a specialty SCAD Clinic at the John Hunter Hospital, Newcastle (R.M.G.), we recorded those cases in our cohort who had a relative with an AD. Participants were asked if they or any relatives had a dissection or aneurysm in any extracoronary artery at any age and recorded the ages and causes of death of grandparents, parents, and siblings. Study participants with a self‐reported family history of AD contacted their relatives about involvement in this study. Twenty‐four relatives consented to participate in the study. Whole‐genome sequencing (WGS) failed for 1 participant. A medical history was recorded via phone interview/medical records (AD cases) or written questionnaire (unaffected family members) for study participants. Additional documentation of AD was obtained for some relatives who had an AD (Figure).

**Figure 1:**
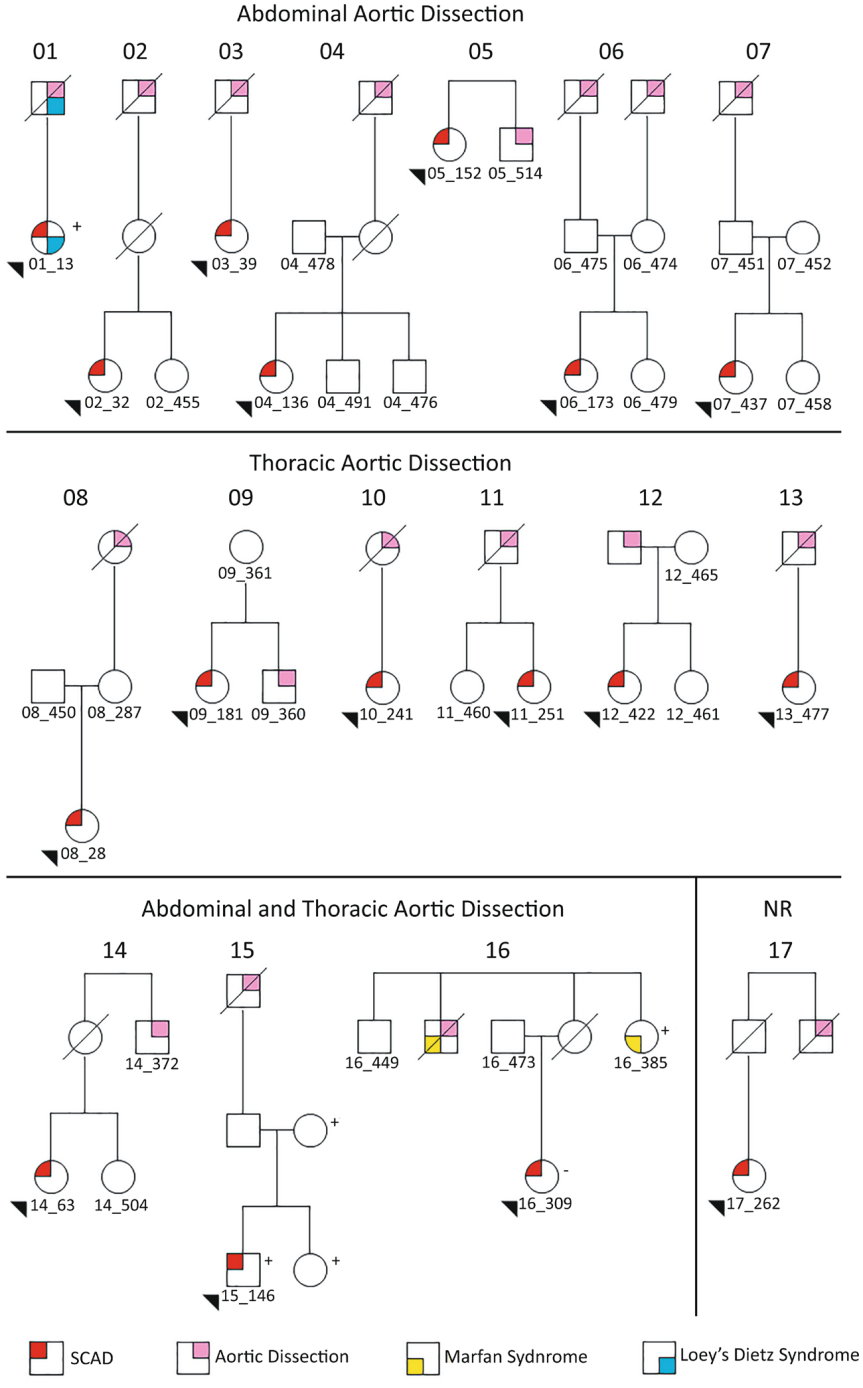
Pedigrees for the SCAD–AD families including affected and sequenced individuals. Arrowheads indicate probands. Women are represented by circles; men are represented by squares. A slash indicates a deceased individual. Phenotype key listed at the bottom. All SCAD diagnoses are angiographically confirmed. AD diagnosis was confirmed through direct interview or medical records for all participants with AD with a sample ID listed. AD was self‐reported by family history for all other individuals with AD except for the grandmother of 08–28 (cardiologist letter), the father of 11_251 (medical records obtained), and the uncle of 16_309 (meticulous medical history notes compiled by 16_385) for whom additional documentation was available. All samples with sample IDs underwent whole‐genome sequencing, with the exception of 01_13. Participant 01_13 and the parents and sister of proband 15_146 had clinical genetic testing. Individuals 01_13 and 16_385 reported their CTD diagnosis, have the physical features of the condition, and carry predicted damaging variants in genes established to be causative for the condition with autosomal dominant inheritance. + indicates that a family member was tested and is heterozygous for a variant linked to a CTD; ‐ indicates that a family member was tested and does not carry the variant; Family 1 + indicates *SMAD3* (p.Met1Val), Family 15 + indicates *COL3A1* p.(Ser934llefsTer35), Family 16 + indicates *FBN1* (chr15:48780559:A>C, predicted splice donor gain). AD indicates aortic dissection; CTD, connective tissue disorder; NR, not reported; and SCAD, spontaneous coronary artery dissection.

All patients with SCAD and relatives who consented for the study provided a mouthwash or blood sample for DNA extraction. Genomic DNA was extracted from buccal cells contained within the mouthwash sample or from whole blood. WGS was performed using Illumina HiSeq X Ten platform with 30× coverage (Kinghorn Centre for Clinical Genomics, Sydney, Australia). Reads were aligned to the GRCh37 reference genome with Burrows–Wheeler Aligner.^20^ Single‐nucleotide variants and insertion/deletion variants were called with the Genome Analysis Toolkit Best Practices pipeline^21^ and multinucleotide variants were joint‐called using Platypus.^22^ Annotations of SNV/insertion/deletion variants were carried out by Annovar^23^ and Variant Effect Predictor,^24^ and included protein‐coding consequences and noncoding region features. Identification of splicing variants was carried out by Spliceogen,^25^ and by using data from SpliceAI^26^ and MMSplice.^27^ Variants were annotated with ClinVar pathogenicity data (version 2022‐11‐05).^28^ Population frequency annotations were from gnomAD (version 2.1.1 and 3.1^29^, ^30^ and gnomAD–multinucleotide variant.)^31^ Structural variants were called using Gridss^32^ and Manta.^33^ Copy number variants were called by CNVnator,^34^ and large copy number variants were called by ConanVarvar.^34^ Structural variants and copy number variants were annotated with region overlaps for protein and regulatory noncoding regions from UCSC Tables,^35^ cohort frequencies, population frequencies from gnomAD‐SV^35^ PEXT heart transcripts,^36^ segmental duplications, and retrocopied gene signatures. Variants were prioritized by VPOT^37^ for manual curation using a minor allele frequency cutoff of 0.1%. Variants were selected for prioritization if they were carried by the SCAD proband cases and the AD cases. Calculation of contamination metrics by VerifyBamID2 (freemix α ≤0.05^38^) and metrics for fragment sizes and chimeric reads (≤5%) by Picard utilities^39^ confirmed that there were no problems in these areas. Relatedness and ethnicity were confirmed, using akt (version 0.3.2).^40^

We analyzed these genomic data for single rare variants classified as likely pathogenic or pathogenic, according to the American College of Medical Genetics and Genomics criteria accessed through Varsome^41^ in tier 1 genes known to associate with aortopathies and CTDs from PanelApp, accessed December 21, 2022.^42^ A broad tier 2 list was then compiled for assessment that included SCAD GWAS^8^ flanking genes, other connective tissue, cardiomyopathy and ion channelopathy, mitral valve prolapse, and Takotsubo and adrenergic genes, as well as genes found in familial SCAD cases^10^ in our cohort (Table S1). We also identified variants classified as pathogenic, likely pathogenic or variants of uncertain significance in ClinVar in tier 1 and tier 2 genes. Furthermore, we performed a genome‐wide assessment for rare variants (absent or with an allele count of <5 in gnomAD population databases of ≈141 000 individuals^30^) in any genes, having pathogenic annotations in ClinVar^28^ for any disease.

We compared the yield of genetic testing in this subcohort of patients with SCAD who had a family history of AD to our cohort of patients with sporadic SCAD.^6^ We selected 17 patients with SCAD with no history of AD and matched them to our current cohort on the basis of sex, age, and age at first SCAD episode.

Polygenic risk for SCAD, FMD, AD, or abdominal aortic aneurysm (AAA) was assessed for SCAD cases and relatives and 1127 controls who were healthy older individuals of European ancestry subjected to WGS.^43^ These controls were from the Medical Genome Reference Bank and were participants from the 45 and Up (59.3% women) and ASPREE (Aspirin in Reducing Events in the Elderly) studies (48.2%).^43^ PRS for AAA was sourced from the Polygenic Score Catalog (ID PGS002054^41^, ^42^, ^44^) while the remaining 3 PRSs were composed of independent, genome‐wide significant variants from the relevant base GWAS: SCAD,^8^ AD,^45^ and FMD.^46^

PRS (as a weighted sum) were calculated using plink (version 1.9)^47^ on the set of PRS variants that were called explicitly in both SCAD–AD and control variant calling pipelines. Variants in regions of high segmental duplication or low complexity were excluded. PRS variants missing from only a small subset of samples were imputed from the rest of the scored sample set. Variants with missingness >0.1 in either call set (SCAD–AD or control) were excluded from all analyses. The association between standardized PRS and disease outcome was analyzed by logistic regression for each of the 4 PRSs (SCAD, FMD, AD, AAA). Standardization used all scored samples (N=1166). Sensitivity analyses were performed using a nonparametric test (Kruskal–Wallis) as well as an unweighted PRS for SCAD (based only on allele risk counts). The distribution of control scores was used to determine percentile thresholds. SCAD–AD family member scores are represented as control percentiles.

## Results

We identified 22 of 435 patients with SCAD who had a self‐reported family history of AD in a first‐ or second‐degree relative, and further investigated 17 of these families. Of these, 16 SCAD probands underwent WGS, and 1 underwent clinical genetic testing. Additionally, WGS was performed on 3 relatives with AD and 20 relatives without a history of SCAD or AD, while 2 additional relatives from family 15 without SCAD or AD underwent clinical genetic testing for a *COL3A1* variant (Figure).

The clinical characteristics of these individuals including a participant with clinical genotyping data are summarized in Table 1. Most of the patients with SCAD were women (16/17 [94.1%]), which reflects the female predominance in our broader cohort and other published cohorts of patients with SCAD. The average age at the time of first SCAD was 48.7 years, similar to other SCAD cohorts. Cases 14_63 and 09_181 had a SCAD recurrence in a different coronary territory. Most cases had a type 2 SCAD (11/17 [64.7%]) or less commonly type 1 (4/17 [23.5%]) or type 3 (2/17 [11.8%]). Medical management only was most common (13/17 [76.5%]), then a percutaneous coronary intervention (4/17 [23.5%]), with 1 of the latter cases going on to coronary artery bypass grafting. FMD was screened for in 13 cases and found in 4 (31%). Extracoronary vascular abnormalities were seen in 4 cases and the aortic root (defined as >2.0 cm/m^2^) and/or ascending aorta (defined as >1.9 cm/m^2^) was mildly dilated in 4 cases on echocardiography.

**Table 1:** Clinical Characteristics of Individuals With SCAD Who Have a Relative With AD

SCAD case number	Sex	Age at SCAD, y	Coronary artery/arteries with SCAD	Angiographic type of SCAD	SCAD management	FMD screening status	Connective tissue disorder	Hypertension	Vascular abnormalities	Aortic root and ascending aorta size
13	F	34	LAD	Type 2	Medical	Screened, no evidence	Loeys–Dietz syndrome type 3	No	Splenic and right internal carotid artery aneurysm	Normal size
32	F	51	LAD	Type 2	Medical	Screened, no evidence	None	No	None	Normal size
39	F	58	LAD	Type 1	Medical	Screened, no evidence	None	No	None	Normal size
136	F	44	LAD	Type 1	Medical	Screened, yes (carotid)	None	No	None	Mildly dilated AsAo
152	F	55	Right PDA	Type 2	Medical	Screened, no evidence	None	No	None	Normal size
173	F	47	LAD	Type 1	PCI	Not screened	None	No	None	Normal size
437	F	44	LAD, LCX – OM1	Type 2	Medical	Screened, no evidence	None	No	None	Normal
28	F	41	LAD, D1	Type 2	PCI	Screened, yes (carotid, renal)	None	No	Carotid dissection	Mildly dilated root, normal AsAo
181	F	59 60	LAD LCX	Type 2	Medical	Screened, yes (carotid, vertebral, renal)	None	Yes	Dilatation of vertebral arteries	Normal size
241	F	59	LAD – D1	Type 2	Medical	Not screened	None diagnosed, clinical features of Ehlers–Danlos/Marfan syndrome	Yes	None	Mildly dilated aortic root
251	F	52	LCX	Type 2	Medical	Screened, no evidence	None	No	None	Normal size
422	F	42	LAD	Type 3	Medical	Screened, no evidence	None	No	None	Normal size
477	F	51	LAD	Type 2	Medical	Not screened	None	No	None	Normal root and mildly dilated AsAo
63	F	46 50	LCX, PL1 Septal branch	Type 2	Medical	Screened, yes (renal)	None	Yes	Cerebral aneurysm, stent to distal left internal carotid	Normal size
146	M	38	RCA	Type 1	PCI and CABG	Screened, no evidence	Vascular Ehlers–Danlos syndrome	No	None	Normal size
309	F	47	Right PDA	Type 2	Medical	Screened, no evidence	None	No	None	Normal size
262	F	60	LAD	Type 3	PCI	Not screened	None	No	None	Normal size

AD indicates aortic dissection; AsAo, ascending aorta; CABG, coronary artery bypass grafting; DI, first diagonal; LAD, left anterior descending; LCX, left circumflex; OM1, first obtuse marginal artery; PCI, percutaneous coronary intervention; PDA, posterior descending artery; PL1, first posterolateral artery; and SCAD, spontaneous coronary artery dissection.

Within the 17 families, all relatives with AD are summarized in Table 2, and the pedigrees are shown in Figure. The first‐ or second‐degree relatives with AD included 4 maternal grandparents, 3 paternal grandparents, 2 maternal uncles, 1 paternal uncle, 2 brothers, 1 mother, and 5 fathers. Of note, 16 of the 18 relatives (89%) with AD were men. The average age of first AD was 68.7 years. The location of the dissection was most commonly abdominal (8/18 [44.4%]), then thoracic (6/18 [33.3%]) followed by thoracic and abdominal (3/18 [1.7%]). In 14 of these relatives, there was an aortic aneurysm associated with the dissection. Four additional participants had an aortic aneurysm; however, there were no cases of participants who had a SCAD and an AD. Fourteen additional study participants in our cohort of 435 had a family history of aortic aneurysms without a dissection. This results in 8.3% of the cohort having a relative with an AD or aortic aneurysm.

**Table 2:** Clinical Details of the Relatives With AD

SCAD case number	Relative with AD	Age of relative at time of AD, y	Location of AD/rupture	Family members with whole‐genome sequencing	Other clinical details for sequenced family members
13	Father	64	Abdominal	None	N/A
32	Maternal grandfather	NR	Abdominal	Sister (455)	None
39	Father	80	Abdominal	None	N/A
136	Maternal grandfather	80	Abdominal	Brother (491)	Mildly dilated aortic root
				Brother (476)	Type 1 diabetes
				Father (478)	None
152	Brother	58	Abdominal	Brother (514)	Abdominal aortic aneurysm and dissection, colon cancer
173	Maternal grandfather	79	Abdominal	Father (475)	Smoker, hepatic artery aneurysm
	Paternal grandfather	98	Abdominal	Mother (474)	Type 2 diabetes
				Sister (479)	
437	Paternal grandfather	87	Abdominal	Father (451)	Ankylosing spondylitis, Hashimoto thyroiditis
				Mother (452)	Migraines, high cholesterol
				Sister (458)	None
28	Maternal grandmother	45	Thoracic	Father (450)	Inflammatory bowel disease, migraines
				Mother (287)	Fibromuscular dysplasia
181	Brother	45	Thoracic	Brother (360)	Thoracic aortic aneurysm and dissection, severe brain damage, quadriplegic
				Mother (361)	Repaired abdominal aneurysm, age 59 y
241	Mother	91	Thoracic	None	N/A
251	Father	40, 46, 53	Thoracic	Sister (460)	None
422	Father	67	Thoracic	Mother (465)	None
				Sister (461)	None
477	Father	72	Thoracic	None	N/A
63	Maternal uncle	61, 65	Thoracic, abdominal	Maternal uncle (372)	Type A and B ADs
				Sister (504)	None
				Father (471)	None
146	Paternal grandfather	83	Thoracic, abdominal	None	N/A
309	Maternal uncle	58	Thoracic, abdominal	Father (473)	None
				Maternal aunt (385)	Mitral valve prolapse + MVR Thoracic aortic aneurysm + AVR and root replacement. Atrial fibrillation. Marfan syndrome
				Other maternal uncle (449)	None
262	Paternal uncle	60	NR	None	N/A

AD indicates aortic dissection; AVR aortic valve replacement; MVR, mitral valve replacement; N/A, not applicable; NR, not reported; and SCAD, spontaneous coronary artery dissection.

Of the 17 patients with SCAD, 3 had single‐nucleotide likely pathogenic or pathogenic variants in tier 1 genes (Table 3). SCAD case 01_13 was heterozygous for a pathogenic variant in *SMAD3* (NM_005902.4:c.1A>G) resulting in a null allele. This patient consequently had a diagnosis of autosomal dominant Loeys–Dietz syndrome type 3. The patient's father with AD had, unfortunately, died at age 64 years but had clinical features of a CTD with hypermobility, scoliosis, translucent skin and easy bruising, mitral valve prolapse and repair, and multiple aneurysms, but was not genetically tested.

**Table 3:** Genetic Results for All Sequenced Family Members Including Pathogenic/Likely Pathogenic and VUSs Found in Tier 1 and Tier 2 Gene Lists and Genome‐Wide

SCAD–AD family	Family member	Nucleotide variant	Gene	Tier	Amino acid variant	ACMG classification	Variant type	gnomAD MAF
13	SCAD case 13*	NM_005902.4:c.1A>G	*SMAD3*	1	p.Met1Val	Pathogenic	Start loss	0
241	SCAD case 241	NM_000071.3:c.919G>A	*CBS*	1	p.Gly307Ser	Pathogenic	Missense	0.000155
146	SCAD case 146	NM_000090.4:c.2798dupG	*COL3A1*	1	p.Ser934llefsTer35	LP	Frameshift	0
146	Mother*	NM_000090.4:c.2798dupG	*COL3A1*	1	p.Ser934llefsTer35	LP	Frameshift	0
146	Sister*	NM_000090.4:c.2798dupG	*COL3A1*	1	p.Ser934llefsTer35	LP	Frameshift	0
39	SCAD case 39	NM_012463.4:c.422G>A	*ATP6V0A2*	1	p.Arg14His	VUS	Missense	0.0000615
63	SCAD case 63	NM_003239.5:c.412T>G	*TGFB3*	1	p.Ser138Ala	VUS	Missense	0.000111
63	Maternal uncle 372 (AD case)	NM_003239.5:c.412T>G	*TGFB3*	1	p.Ser138Ala	VUS	Missense	0.000111
63	Sister 504	NM_003239.5:c.412T>G	*TGFB3*	1	p.Ser138Ala	VUS	Missense	0.000111
39	SCAD case 39	NM_006933.7:c.1939G>T	*SLC5A3*	2	p.Glu647Ter	VUS	Stop‐gain	0
152	SCAD case 152	NM_019032.6:c.2398G>T	*ADAMTSL4*	2	p.Gly800Cys	VUS	Missense	0
152	Brother 514 (AD case)	NM_019032.6:c.2398G>T	*ADAMTSL4*	2	p.Gly800Cys	VUS	Missense	0
39	SCAD case 39	NM_144573.4:c.201G>A	*NEXN*	N/A	p.Trp67Ter	Pathogenic	Stop‐gain	0
39	SCAD case 39	NM_004820.5:c.650dup	*CYP7B1*	N/A	p.Leu217PhefsTer4	Pathogenic	Frameshift	0.00000798
28	SCAD case 28	NM_001130987.2:c.5830C>T	*DYSF*	N/A	p.Arg1944Ter	Pathogenic	Stop‐gain	0.0000119
28	Mother 287	NM_001130987.2:c.5830C>T	*DYSF*	N/A	p.Arg1944Ter	Pathogenic	Stop‐gain	0.0000119
251	SCAD case 251	NM_000274.4:c.734A>G	*OAT*	N/A	p.Tyr245Cys	LP	Missense	0.00000398
251	Sister 460	NM_000274.4:c.734A>G	*OAT*	N/A	p.Tyr245Cys	LP	Missense	0.00000398

AAA indicates abdominal aortic aneurysm; ACMG, American College of Medical Genetics and Genomics; AD, aortic dissection; FMD, fibromuscular dysplasia; LP, likely pathogenic; SCAD, spontaneous coronary artery dissection; and VUS, variant of uncertain significance.

* Cases had clinical sequencing only.

In SCAD case 10_241, the patient was heterozygous for a pathogenic variant in *CBS* (NM_000071.3:c.919G>A), resulting in amino acid variant p.(Gly307Ser). The patient's mother with AD had died, and no other family member consented to genetic testing.

In SCAD case 15_146, the patient was heterozygous for a likely pathogenic variant in *COL3A1* (NM_000090.4:c.2798dupG) resulting in amino acid variant p.(Ser934llefsTer35), as previously reported.^6^ The patient's paternal grandfather with AD was deceased and thus was unable to be tested; however, his mother and sister were positive for the variant and his father was negative. The patient was diagnosed with autosomal dominant vascular Ehlers–Danlos syndrome but, aside from easy bruising, did not have other features of the condition on careful phenotypic assessment.

Overall, we identified a pathogenic or likely pathogenic variant in 3 of 17 patients with SCAD (17.6%) with a family history of an AD. Despite our small sample size, this indicates a potentially higher diagnostic rate for this cohort compared with our cohort of patients with sporadic SCAD (11%).^6^ To ensure a fair comparison, we selected 17 patients with SCAD with no history of AD from our previously analyzed cohort of 91 patients with sporadic SCAD.^6^ These 17 cases were matched with the current cohort on the basis of sex, age, and age at first SCAD episode. Among these, only 2 individuals were carriers of pathogenic or likely pathogenic variants, yielding a diagnostic rate of ≈12%.

There were 4 further variants of uncertain significance (VUS) in tier 1 and 2 genes. In SCAD case 03_39, the patient was homozygous for a VUS with a PP3 criterion (computational evidence supporting pathogenicity) strong pathogenic in *SLC5A3* (NM_006933.7:c.1939G>T) resulting in premature protein termination p.(Glu647Ter). Case 03_39 also had a heterozygous variant, NM_012463.4:c.422G>A, classified as a VUS in ClinVar (but as likely benign by Varsome) in *ATP6V0A2* resulting in a p.(Arg14His). The patient's father with AD had died, and no other family member consented to genetic testing. In SCAD case 05_152, the patient and her brother with abdominal AD were heterozygous for a VUS with a PP3 strong pathogenic in *ADAMTSL4* (NM_019032.6:c.2398G>T) resulting in amino acid variant p.(Gly800Cys). The patient in case 14_63, her maternal uncle with AD, and her unaffected sister had a heterozygous variant in *TGFB3*, NM_003239.5:c.412T>G, classified as a VUS on ClinVar for Rienhoff syndrome (Loeys–Dietz syndrome 5) resulting in a p.Ser138Ala. Two additional patients were reported in ClinVar with this variant and a history of AD (1 classified as a VUS).

Genome‐wide assessment for variants in genes other than the tier 1 and 2 genes, detected 4 heterozygous variants classified as likely pathogenic/pathogenic variants in ClinVar for other diseases (Table 3). A variant in *NEXN* (NM_144573.4:c.201G>A) in case 03_39, resulted in stop‐gain (p.Trp67Ter). This variant was classified as pathogenic for dilated cardiomyopathy and hypertrophic cardiomyopathy and is possibly associated with a spectrum of diseases. Loss‐of‐function variants in *NEXN* are known to be pathogenic and the gene has both autosomal dominant and recessive modes of inheritance with variable penetrance.^48^, ^49^, ^50^ Notably, this was the only one of these 4 variants associated with a disease showing potential autosomal dominant inheritance. A variant in *CYP7B1* (NM_004820.5:c.650dup) in case 03_39, resulted in a frameshift (p.Leu217PhefsTer4). The mode of inheritance of *CYP7B1* variants is autosomal recessive for spastic paraplegia^51^ and in these cases leads to a premature translational stop signal. A variant in *DYSF* (NM_001130987.2:c.5830C>T) in case 08_28 and her affected mother 08_287, resulted in a stop‐gain (p.Arg1944Ter). This variant has been reported in the literature as a biallelic genotype in multiple individuals affected by limb‐girdle muscular dystrophy or Miyoshi myopathy. The mode of inheritance is autosomal recessive for muscular dystrophies.^52^ A variant in *OAT* (NM_000274.4:c.734A>G) was found in case 11_251 and her unaffected sister 11_460, resulting in amino acid change (p.Tyr245Cys). This variant has been found in individuals with gyrate atrophy, and the mode of inheritance is autosomal recessive.^53^

Genome‐wide structural variant analysis did not reveal any pathogenic or likely pathogenic variants in the tier 1 or 2 genes; however, 1 variant of uncertain significance was identified (chr10:79136316:79136399:DEL) in *KCNMA1* in case 03_39.

Polygenic risk scores for AAA, AD, SCAD, or FMD were calculated for 16 patients with SCAD, 3 patients with AD, and 20 relatives without SCAD or AD, giving a total of 39 family members with SCAD–AD, and 1127 healthy controls (Table S2). Due to the scarcity of individuals in these families with AD, and the heterogeneity in perceived risk/inheritance for unaffected family members, only patients with SCAD and control individuals were included in logistic regressions. A PRS for SCAD was associated with higher odds of having experienced a SCAD compared with the healthy controls (Tables S2; Figure S1). With every SD increase in PRS_SCAD_, the odds of having experienced a SCAD were 1.79 times higher (95% CI, 1.08–2.99; *P*=0.024). Sensitivity analyses using a nonparametric test (Kruskal–Wallis χ^2^=3.9764, df=1; *P*=0.04614) and an unweighted PRS (OR, 1.26 [95% CI, 1.03–1.55]; *P*=0.0237) confirmed this association. There was a trend toward association between SCAD status and PRS for FMD, although this was not significant (95% CI, 0.99–2.88; *P*=0.058). No such pattern was detectable using PRS for AD or AAA (Table S2; Figure S1). Scores for all family members, as a percentile of the scores in the healthy controls are given in Table S3.

## Discussion

We report the largest series of patients with SCAD with a family history of AD described to date. AD is a rare event, occurring at an overall incidence of 6 per 100 000 in a large population‐based study, but this varies with age and sex; men have a higher incidence at all ages, and both sexes have a steeper increase in incidence from age 60 years.^54^ The increased incidence in men was observed in our data, with 89% of relatives with AD being men. Compared with general population data,^54^ the male:female ratio is higher in our cohort, and the age of onset is lower. This sex‐specific manifestation could be attributed to complex interactions between genetic factors and sex‐specific physiological elements, such as hormonal influences or differential gene expression patterns.

The clustering of these traits, younger age of onset of AD, and pathogenic similarities of SCAD and AD raises the possibility of a shared genetic predisposition between the 2 conditions, at least in some families. We found a higher diagnostic yield of genetic testing in this subcohort of patients with SCAD with a relative who had AD compared with matched (sex, age, and age at first SCAD) controls with SCAD. While based on small sample sizes and slightly different candidate gene lists, this finding supports the notion that patients with SCAD with a family history of AD may have a higher diagnostic yield.

Clinically, 4 of the 17 patients with SCAD had a mildly dilated aortic root or ascending aorta, and 2 of these cases had a confirmed diagnosis of FMD (the other 2 were not screened). A study using computed tomography angiography to assess the aortic dimensions of female patients with a diagnosis of FMD compared with a comparison group reported that patients with FMD had a larger aortic dimension, most pronounced at the sinotubular junction and ascending aorta.^55^ The increase in aortic dimensions in a portion of our cohort could represent an association with SCAD, especially in patients who have a family history of AD, or an association with FMD, as previously reported.^55^

There were several likely pathogenic/pathogenic SNVs identified in genes associated with arterial dissection. One of our patients (case 01_13) was heterozygous for a pathogenic variant in *SMAD3* (NM_005902.4:c.1A>G) resulting in a null allele and a diagnosis of Loeys–Dietz syndrome. A *SMAD3* variant (c.860G>A) has been reported previously in a SCAD–AD family, involving a 34‐year‐old female proband who experienced 5 SCADs.^56^ Several years later, she presented with a type A AD, and her 41‐year‐old brother also presented with a left anterior descending coronary artery SCAD. The proband also had 2 uncles, not confirmed to carry the variant, who died at age 40 years due to AD.^56^ In case 01_13, it is plausible that the pathogenic variant in *SMAD3* contributed to a large extent to developing the dissection; however, in other families where relatives have been genetically screened, as is the case for families 14 and 15, there are family members who carry the variant linked to dissection but have yet not experienced a dissection event. It is possible that these are predisposing alleles, rather than highly penetrant pathogenic alleles.

*COL3A1* is the most reported monogenic candidate gene in SCAD, with at least 21 unique cases with likely pathogenic/pathogenic *COL3A1* variants in the literature,^57^ inclusive of our case. Variants in *COL3A1* are associated with vascular Ehlers–Danlos syndrome, but interestingly, our case (15_146), similar to other cases of SCAD with *COL3A1* variants described in the literature, did not have the typical phenotypic features of this CTD. The SCAD proband's mother, father, and sister were tested for the *COL3A1* variant; the sister and mother were heterozygous for the variant but have not suffered dissection events, and the father was negative for the variant. This was despite the paternal grandfather having died of AD, so it is possible that 15_146 inherited independent genetic risk from both sides of his family. Unlike family 01, in which the dissections assorted with the CTD, in family 16 the observed dissections occurred in 1 family member with Marfan syndrome and 1 without. Only 1 family member (16_385) who was tested had a Marfan syndrome diagnosis and did not have a dissection. No predicted damaging variants were identified in the coding regions of *FBN1* (known to cause Marfan syndrome when mutated); however, a novel variant was found that is predicted to alter splicing in *FBN1* (chr15:48780559:A>C, predicted splice donor gain), which was not carried by the SCAD proband. It is possible that this family also has ≥2 variants that predisposed them to dissections as observed in 2 families with Marfan syndrome and aortic aneurysms or ADs assorting independently.^58^

We identified a patient with SCAD (case 10_241) with a *CBS* variant, a gene that encodes for cystathionine β‐synthase and is typically associated with autosomal recessive phenotypes including a Marfan‐like syndrome and homocystinuria.^59^ Our patient was heterozygous for a pathogenic variant in *CBS*, and there is strong evidence linking this gene to AD. There are also 2 published cases of elevated homocysteine levels being associated with SCAD.^60^, ^61^ In addition, in a cohort of patients with SCAD who underwent genetic testing, 4 cases with 4 unique VUSs (as per the authors' determination) in *CBS* were identified, with 1 of those VUSs classified as pathogenic by Varsome's current American College of Medical Genetics and Genomics analysis (NM_000071:c.502G>A, p.Val168Met). The patient with SCAD carrying this variant was not reported to have a clear phenotype of homocystinuria, and it is unclear if these cases had a family history of dissections.^62^

The VUS SNVs have some evidence to support the involvement of those genes with SCAD. A locus in *ADAMTSL4* correlated with SCAD in the meta‐GWAS and accounted for the largest proportion of heritability for SCAD.^8^ A locus in *SLC5A3* was also correlated with SCAD.^8^ *TGFB3* is a tier 1 gene from the PanelApp aortopathy and CTD gene panel and is associated with autosomal dominant Loeys–Dietz syndrome 5 and aortic aneurysm.^42^

Four variants were found in genes that are not currently associated with SCAD or AD but are pathogenic for dilated cardiomyopathy, hypertrophic cardiomyopathy, spastic paraplegia, and ornithine aminotransferase deficiency. The patients are heterozygous for these variants, but most of these diseases are thought to show autosomal recessive inheritance. Hence, these variants could be incidental findings of the samples' carrier status. However, given our limited knowledge of the genetics of SCAD and AD, we report these variants here to facilitate future investigation on additional stress mechanisms that could be involved in SCAD and AD, as well as other arterial dissections.

A PRS using the 16 recently identified SCAD loci was found to associate with a higher odds of SCAD, compared with controls, supporting our previous report based on a smaller SCAD PRS, consisting of only 7 loci.^10^ There was no clear pattern in the other PRS for AAA, AD, or FMD potentially due to the limited sample size of our cohort.

## Limitations

One limitation of our study is that despite, to our knowledge, this being the largest reported series of familial clustering of SCAD and AD, the number of patients and relatives with AD is small, and in 14 of 18 cases the AD‐affected relatives were deceased, which led to difficulties obtaining clinical information, such as hospital medical records, relevant to their AD (type A versus abdominal). This is primarily due to the high mortality rate associated with AD, which limited our power to identify and segregate rare variants with dissections within pedigrees, and to draw broader cohort‐wide conclusions from the PRS results.

For PRS analyses, the small sample size is a significant limitation, as it increases the influence of individual data points. One individual with a high SCAD PRS value, visible in Figure S1, influences the results. However, this value lies within the control distribution and aligns with prior findings in studies of polygenic risk in SCAD. Therefore, we do not consider it an outlier, but rather an expected observation within the inherent variability of small data sets. This underscores the need for larger cohort studies to validate these findings and reduce the impact of individual variability on statistical analyses.

Participants in this study with SCAD were recruited through social media posts and word of mouth, which may have introduced some selection bias. Individuals with comorbidities or a family history of cardiovascular events may have been more inclined to participate, potentially inflating the proportion of patient with SCAD with a family history of AD, making it less representative of the general population.

Additionally, not all patients with SCAD in this study underwent comprehensive screening for FMD or thorough evaluation for aortic disease, which limits our ability to accurately assess the incidence of FMD and aortic pathology in this cohort.

## Conclusions

In this study involving the largest clustering of familial SCAD and AD reported to date, the genetics, potentially underpinning the clustering, suggests a role for both rare pathogenic variants and polygenic risk. Pathogenic variants in *SMAD3* and *CBS* were identified in 2 patients with SCAD with an AD‐affected relative. Several single‐nucleotide and structural variants of uncertain significance in tier 1 and tier 2 SCAD genes were also identified, which may pave the way for future replication in other AD cohorts. Polygenic risk scores using 16 recently identified SCAD loci were found to be associated with a higher risk of SCAD in this cohort of patients who had a relative with AD. Whether the results from patients with SCAD who have a close AD‐affected relative represent pleiotropy, multiple risk alleles common to both conditions, or independently assorting genetically unrelated phenotypes, remains to be fully determined. However, these results, which hint at the shared genetics of these conditions, suggest further research is warranted.

## Sources of Funding

This work was supported in part by grants from the Cardiac Society of Australia and New Zealand, the National Health and Medical Research Council, Australia (APP1161200), the St Vincent's Clinic Foundation, the Catholic Archdiocese of Sydney, Perpetual Philanthropy, NSW Health, and SCAD Research Inc. E.G. is supported by a New South Wales Health Early Mid‐Career Cardiovascular Grant and a National Health and Medical Research Council Investigator Grant (2018360). J.C.K. acknowledges research support from the US National Institutes of Health (R01HL148167, R01HG012773), New South Wales Health grant RG194194, the Bourne Foundation, Snow Medical, and Agilent. R.M.G. is supported by an National Health and Medical Research Council L3 Investigator Grant (APP2010203) and a New South Wales Health Cardiovascular Senior Scientist Grant. L.M.C. is supported by a National Health and Medical Research Council Postgraduate Scholarship (GNT2013809) with cofunding from a National Heart Foundation PhD Scholarship (106228).

## Disclosures

The authors have no disclosures to report.

## Supporting information

Tables S1–S3
Figure S1
