# Supplementary material for: Spontaneous Coronary Artery Dissection and a Family History of Aortic Dissection: A Genetic Association Study
Source: J Am Heart Assoc. 2025 Apr 7;14(8):e037921. doi: 10.1161/JAHA.124.037921 (PMC12132886; doi:10.1161/JAHA.124.037921)
Supplement: Supplementary file 1 — Tables S1–S3 Figure S1 [file JAH3-14-e037921-s001.pdf]

Supplemental Material

Table S1: Tier 1 and Tier 2 genes including presence or absence on PanelApp.

Gene	Tier	PanelApp	PanelApp confidence rating
ABL1	Tier 1	Yes	Green
ACTA2	Tier 1	Yes	Green
ADAMTS10	Tier 1	Yes	Green
ADAMTS17	Tier 1	Yes	Green
ADAMTS2	Tier 1	Yes	Green
AEBP1	Tier 1	Yes	Green
ALDH18A1	Tier 1	Yes	Green
ARIH1	Tier 1	Yes	Green
ATP6V0A2	Tier 1	Yes	Green
ATP6V1E1	Tier 1	Yes	Green
ATP7A	Tier 1	Yes	Green
B3GALT6	Tier 1	Yes	Green
B4GALT7	Tier 1	Yes	Green
BGN	Tier 1	Yes	Green
C1R	Tier 1	Yes	Green
C1S	Tier 1	Yes	Green
CBS	Tier 1	Yes	Green
CHST14	Tier 1	Yes	Green
COL11A1	Tier 1	Yes	Green
COL12A1	Tier 1	Yes	Green
COL1A1	Tier 1	Yes	Green
COL1A2	Tier 1	Yes	Green
COL2A1	Tier 1	Yes	Green
COL3A1	Tier 1	Yes	Green
COL5A1	Tier 1	Yes	Green
COL5A2	Tier 1	Yes	Green
DLG4	Tier 1	Yes	Green
DSE	Tier 1	Yes	Green
EFEMP1	Tier 1	Yes	Green
EFEMP2	Tier 1	Yes	Green
ELN	Tier 1	Yes	Green
EMILIN1	Tier 1	Yes	Green
FBLN5	Tier 1	Yes	Green
FBN1	Tier 1	Yes	Green
FBN2	Tier 1	Yes	Green
FKBP14	Tier 1	Yes	Green

FLNA	Tier 1	Yes	Green
IPO8	Tier 1	Yes	Green
KCNMA1	Tier 1	Yes	Green
LOX	Tier 1	Yes	Green
LTBP1	Tier 1	Yes	Green
LTBP3	Tier 1	Yes	Green
LTBP4	Tier 1	Yes	Green
MED12	Tier 1	Yes	Green
MYH11	Tier 1	Yes	Green
MYLK	Tier 1	Yes	Green
NOTCH1	Tier 1	Yes	Green
NPR3	Tier 1	Yes	Green
PCGF2	Tier 1	Yes	Green
PDGFRB	Tier 1	Yes	Green
PLOD1	Tier 1	Yes	Green
PLOD3	Tier 1	Yes	Green
PRDM5	Tier 1	Yes	Green
PRKG1	Tier 1	Yes	Green
PTDSS1	Tier 1	Yes	Green
PYCR1	Tier 1	Yes	Green
RIN2	Tier 1	Yes	Green
SKI	Tier 1	Yes	Green
SLC2A10	Tier 1	Yes	Green
SLC39A13	Tier 1	Yes	Green
SMAD2	Tier 1	Yes	Green
SMAD3	Tier 1	Yes	Green
SMAD4	Tier 1	Yes	Green
TGFB2	Tier 1	Yes	Green
TGFB3	Tier 1	Yes	Green
TGFBR1	Tier 1	Yes	Green
TGFBR2	Tier 1	Yes	Green
THSD4	Tier 1	Yes	Green
TNXB	Tier 1	Yes	Green
ZNF469	Tier 1	Yes	Green
ADAMTSL2	Tier 1	Yes	Amber
ATP6V1A	Tier 1	Yes	Amber
COL6A1	Tier 1	Yes	Amber
COL6A3	Tier 1	Yes	Amber
FOXE3	Tier 1	Yes	Amber
GORAB	Tier 1	Yes	Amber
HCN4	Tier 1	Yes	Amber
JAG1	Tier 1	Yes	Amber

MAPK8	Tier 1	Yes	Amber
MAT2A	Tier 1	Yes	Amber
MFAP5	Tier 1	Yes	Amber
PIEZO2	Tier 1	Yes	Amber
ROBO4	Tier 1	Yes	Amber
SMAD6	Tier 1	Yes	Amber
TLN1	Tier 1	Yes	Amber
ABCC6	Tier 1	Yes	Red
COL6A2	Tier 1	Yes	Red
HEY2	Tier 1	Yes	Red
P4HA1	Tier 1	Yes	Red
PI4K2A	Tier 1	Yes	Red
HSD52	Tier 2	No	N/A
FGGY	Tier 2	No	N/A
F3	Tier 2	No	N/A
SLC44A3-AS1	Tier 2	No	N/A
ECM1	Tier 2	No	N/A
ADAMTSL4	Tier 2	No	N/A
AFAP1	Tier 2	No	N/A
ZNF827	Tier 2	No	N/A
ITGA1	Tier 2	No	N/A
PHACTR1	Tier 2	No	N/A
HTRA1	Tier 2	No	N/A
SESN3	Tier 2	No	N/A
FAM76B	Tier 2	No	N/A
LRP1	Tier 2	No	N/A
POC1B	Tier 2	No	N/A
ATP2B1	Tier 2	No	N/A
COL4A1	Tier 2	No	N/A
COL4A2	Tier 2	No	N/A
MRPS6	Tier 2	No	N/A
ADAMTSL4	Tier 2	No	N/A
KCNE2	Tier 2	No	N/A
SYN3	Tier 2	No	N/A
TIMP3	Tier 2	No	N/A
CACNA1C	Tier 2	No	N/A
ABCB1	Tier 2	No	N/A
HDLBP	Tier 2	No	N/A
LTBP2	Tier 2	No	N/A
KCNJ2	Tier 2	No	N/A
ERBB4	Tier 2	No	N/A
FLNC	Tier 2	No	N/A

LMNA	Tier 2	No	N/A
CRTAP	Tier 2	No	N/A
P3H1	Tier 2	No	N/A
B3GAT1	Tier 2	No	N/A
SH3PXD2B	Tier 2	No	N/A
TNS1	Tier 2	No	N/A
LMCD1	Tier 2	No	N/A
TGM2	Tier 2	No	N/A
BAG3	Tier 2	No	N/A
ADRB1	Tier 2	No	N/A
GRK5	Tier 2	No	N/A
ADRB1	Tier 2	No	N/A
ADRB2	Tier 2	No	N/A

Table S2: Association between SCAD status and polygenic risk scores for SCAD, FMD (Fibromuscular Dysplasia), AD (Aortic Dissection) and AAA (abdominal aortic aneurysm).

**PRS**	**OR (95% CI)**	***P* value**
SCAD	1.79 (1.08-2.99)	0.024
FMD	1.67 (0.99 – 2.88)	0.058
AD	1.01 (0.63 – 1.69)	0.977
AAA	1.14 (0.69 – 1.88)	0.614

Table S3: Percentiles of polygenic risk scores for individuals with SCAD and sequenced family members. Percentiles are calculated based on Medical Genome Reference Bank (MGRB) controls. PRS, polygenic risk score; SCAD, spontaneous coronary artery dissection; AD, aortic dissection; AAA, abdominal aortic aneurysm.

SCAD-AD family	Family member	PRS SCAD (%)	PRS AAA (%)	PRS AD (%)	PRS FMD (%)
02	SCAD case 02_32	51	47	63	68
02	Sister 02_455	16	21	18	63
03	SCAD case 03_39	64	58	4	38
04	SCAD case 04_136	89	63	99	98
04	Brother 04_491	83	65	63	77
04	Brother 04_476	94	46	99	98
04	Father 04_478	55	49	63	91
05	SCAD case 05_152	75	87	63	99
05	Brother 05_514 (AD case)	70	50	63	99
06	SCAD case 06_173	96	72	99	98
06	Father 06_475	74	83	86	71
06	Mother 06_474	83	50	70	89
06	Sister 06_479	59	87	70	98
07	SCAD case 07_437	55	3	63	77
07	Father 07_451	78	5	63	60
07	Mother 07_452	52	48	63	99
07	Sister 07_458	65	0	99	77
08	SCAD case 08_28	99	78	22	91
08	Father 08_450	79	44	40	70
08	Mother 08_287	82	81	40	40
09	SCAD case 09_181	53	93	47	93
09	Brother 09_360 (AD case)	89	94	70	77
09	Mother 09_361	84	92	99	91
10	SCAD case 10_241	66	28	13	22
11	SCAD case 11_251	40	60	70	77
11	Sister 11_460	27	4	70	77
12	SCAD case 12_422	82	5	99	77
12	Mother 12_465	95	13	99	77
12	Sister 12_461	95	23	99	91
13	SCAD case 13_477	91	31	86	53
14	SCAD case 14_63	32	32	2	22
14	Maternal uncle 14_372 (AD case)	70	18	63	62
14	Sister 14_504	96	24	8	93
15	SCAD case 15_146	85	74	63	26
16	SCAD case 16_309	30	93	18	6
16	Father 16_473	62	77	40	22
16	Maternal uncle 16_449	88	35	47	53
16	Maternal aunt 16_385	44	73	86	40
17	SCAD case 17_262	17	50	52	63

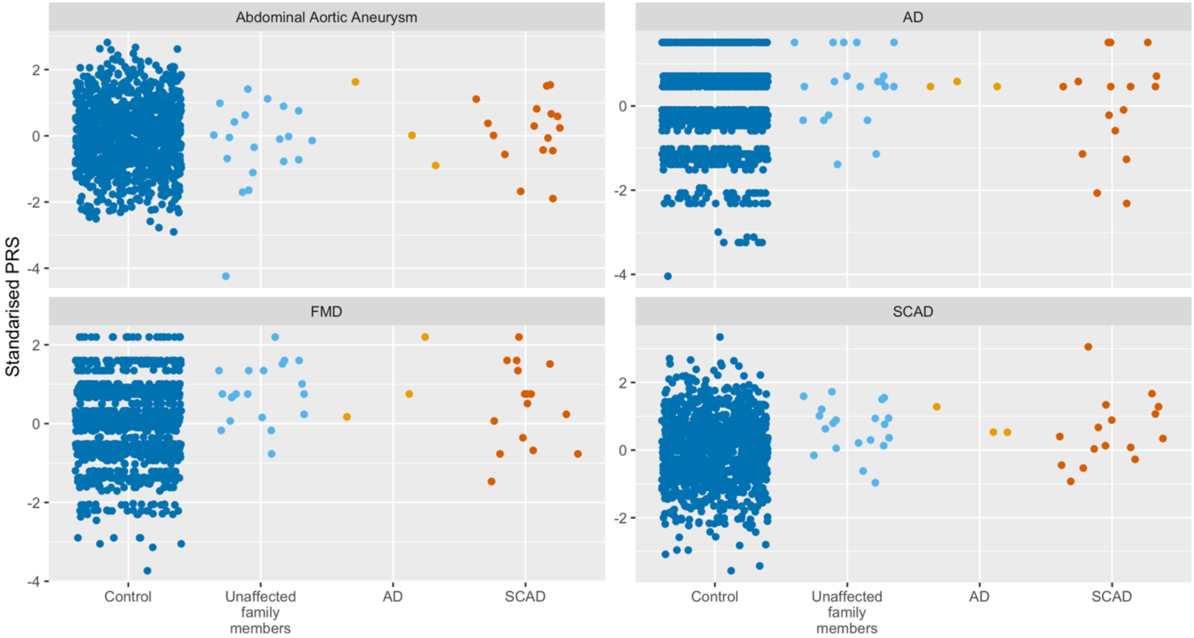

Figure S1: Standardised Polygenic Risk Scores for SCAD, FMD (Fibromuscular Dysplasia), AD (Aortic Dissection) and AAA (abdominal aortic aneurysm) shown by disease status.
